# Requirements and challenges of hospital dashboards: a systematic literature review

**DOI:** 10.1186/s12911-022-02037-8

**Published:** 2022-11-08

**Authors:** Reza Rabiei, Sohrab Almasi

**Affiliations:** Department of Health Information Technology and Management, School of Allied Medical Sciences, Shahid Beheshti University of Medical Sciences, Darband St, Qods Square, Shariati St, Tehran, Iran

**Keywords:** Hospital, Dashboard, Capabilities, Challenge

## Abstract

**Background:**

Today, the use of data in administrative and clinical processes is quite challenging due to the large volume of data, data collection from various sources, and lack of data structure. As a data management tool, dashboards play an important role in timely visual display of critical information on key performances.

**Objectives:**

This systematic review aimed to identify functional and non-functional requirements, as well as challenges of using dashboards in hospitals.

**Methods:**

In this systematic review, four databases, including the Web of Science, PubMed, EMBASE, and Scopus, were searched to find relevant articles from 2000 until May 30, 2020. The final search was conducted on May 30, 2020. Data collection was performed using a data extraction form and reviewing the content of relevant studies on the potentials and challenges of dashboard implementation.

**Results:**

Fifty-four out of 1254 retrieved articles were selected for this study based on the inclusion and exclusion criteria. The functional requirements for dashboards included reporting, reminders, customization, tracking, alert creation, and assessment of performance indicators. On the other hand, the non-functional requirements included the dashboard speed, security, ease of use, installation on different devices (e.g., PCs and laptops), integration with other systems, web-based design, inclusion of a data warehouse, being up-to-data, and use of data visualization elements based on the user’s needs. Moreover, the identified challenges were categorized into four groups: data sources, dashboard content, dashboard design, implementation, and integration in other systems at the hospital level.

**Conclusion:**

Dashboards, by providing information in an appropriate manner, can lead to the proper use of information by users. In order for a dashboard to be effective in clinical and managerial processes, particular attention must be paid to its capabilities, and the challenges of its implementation need to be addressed.

**Supplementary Information:**

The online version contains supplementary material available at 10.1186/s12911-022-02037-8.

## Introduction

Today, healthcare organizations, including hospitals, are struggling with different sources of information chaos, such as information overload/underload, erroneous information, information scatter, and information conflict. Information chaos not only causes dissatisfaction, fatigue, and disappointment among healthcare providers, but also exerts negative effects on patient safety [1]. On the other hand, the use of data in managerial and clinical processes is quite challenging due to the large volume of data, data collection from various sources, and lack of data structures. Consequences, such as increased errors [2], delayed care delivery [3], and reduced patient safety [4] are experienced due to poor data management and presentation.

Since hospitals provide a broad spectrum of diagnostic, curative, and administrative services in a complex and dynamic environment, there is an urgent need for continuous performance monitoring in different hospital departments for proper resource management and high-quality healthcare delivery [5]. Therefore, it is necessary for healthcare providers to obtain the necessary information using a comprehensive and organized method [6]. In fact, dashboards are data management tools that collect data from various information systems available in the organization and present it in a concise, comprehensive, meaningful, and intelligent manner in the form of key performance indicators with alerts on the status of these indicators. In this way, managers can briefly evaluate the performance of their department, identify the problems, and analyze their roots to improve their performance. This process allows managers to briefly evaluate the performance of their departments, identify the existing problems, and analyze their root causes to improve their performance [7, 8].

By providing accurate and timely information, dashboards are not only effective systems to meet the information needs of organizations, but are also helpful in the management of large amounts of data in these organizations [9]. The use of quality dashboards in healthcare settings is expanding. Healthcare dashboards are divided into two main categories of clinical and quality dashboards. Clinical dashboards provide timely and relevant information to help decision-making about patients and improve care [10, 11], whereas quality dashboards provide managers with key performance indicators at the department or organization level to help decision-making [12, 13].

Both functional and non-functional requirements need to be considered when developing a dashboard. The functional requirements deal with functions that a system is intended to perform or deliver [14]. On the other hand, non-functional requirements are a set of specifications that describe the system’s operation capabilities and constraints and attempt to improve its functionality. These are basically the requirements that outline how well it will operate including things like speed, security, reliability, data integrity, etc. [14, 15].

The results of studies show that the use of dashboards have the potential to accelerate data collection, reduce cognitive burden, reduce errors, and improve the awareness situation in healthcare settings [16–18]. The review by Dowding et al. showed that there is some evidence that using dashboards that provide immediate access to information for clinicians can improve adherence to quality guidelines and may help improve patient outcomes [19].

Despite the increasing use of dashboards in hospitals and other health care settings, there are still challenges in content, design, implementation, and integration with other systems. Although there has been research on dashboards in health care, to our knowledge, no systematic review has been conducted on functional and non-functional requirements as well as challenges of hospital dashboards. The current study aimed to review challenges with hospital dashboards and provide recommendations for improvement.

## Methods

### Data sources and search strategy

The present study was conducted based on the Preferred Reporting Items for Systematic Reviews and Meta-Analyses (PRISMA) statement [20]. The Web of Science, PubMed, EMBASE, and Scopus databases were searched to identify relevant studies. In the search strategy, combinations of MeSH terms, Emtree terms, and keywords related to dashboards, Capabilities, and hospital were used (Table 1). The search was conducted on May 30, 2020. One researcher (SA) searched the literature and retrieved relevant studies independently. Any uncertainty with the other author (RR) were discussed and resolved. The final stage of the search strategy was the bibliographic check of the selected articles.

**Table 1:** Search strategy and keywords

1#	dashboard OR whiteboard OR status board OR Electronic tracking board OR visualization OR presentation format OR display format OR performance measurement system
2#	Design OR capability OR feature OR character OR attributes OR function OR usability OR content
3#	Hospital
AND	1# AND 2# AND 3#

### Inclusion and exclusion criteria

The inclusion criteria were as follows: (1) articles written in English; (2) articles that focused on quality/clinical dashboard implementation at hospitals or in hospital wards; and (3) articles that addressed functional and non-functional requirements and challenges of implementing dashboards. The exclusion criteria were non-English articles and studies investigating the implementation of clinical/quality dashboards in non-hospital settings.

### Study selection, appraisal, and data extraction

The eligible articles were identified in a two stage process: (1) screening the study title and abstract; and (2) selection of articles by reviewing the full-text manuscripts. Two researchers (RR and SA) independently evaluated the retrieved articles based on the inclusion and exclusion criteria. Each reviewed article was classified as undecided, excluded, or included. The undecided articles were discussed by both reviewers, and then they performed quality assessments independently, and any discrepancies were resolved in group discussions. The Cochrane Effective Practice and Organization of Care (EPOC) guidelines were used to evaluate the quality of retrieved articles. This guideline is developed for the quality assessment of clinical trials, non-clinical trials, before-and-after trials, and case studies [21].

In the data extraction stage, the year of publication, country, and setting were documented for each article (Figs. 2, 3, 4). Next, the goals and requirements of dashboards were categorized as functional requirements, non-functional requirements, and system applications (Table 2). Besides, challenges were categorized into four groups based on similar studies: data source and data generation, dashboard content, dashboard design, and implementation and integration (Table 3).

**Table 2:** Goals and requirements for a hospital dashboard

Dashboard’s application and requirements	Studies
*System applications*	
Quality of care assessment	[24, 26, 28–31, 39, 48–51, 53–55, 57, 59–62, 65, 66, 74]
Resource management	[25, 28, 52, 71]
Activities monitoring	[22, 24–31, 36, 38, 45–66, 68, 70–72, 74]
Analysis and forecasting	[30–32, 48, 65, 68]
*Functional requirements*	
Customization	[26–44, 50–52, 57–60, 62–66, 68–74]
Alert creation	[23, 26, 28–30, 33, 35–38, 40–45, 47–49, 53, 54, 56–60, 63–66, 68–70, 72, 73]
Tracking	[28, 33, 43–49, 53, 54, 56, 57]
Performance indicators measurement	[27, 31, 36, 39, 44, 45, 48, 57, 59–62, 69, 71, 72]
Reporting	[22–49, 53–55, 57–72, 74]
Reminder messages	[26, 29]
*Non-functional requirements*	
Use data visualization elements to display data based on user needs	[18, 24, 27–45, 57, 58, 61–64, 66–74]
Ability to install on a variety of devices such as PC and Laptop	[25, 49, 58, 69]
Speed	[28, 36, 43, 69]
Integration with other systems	[26, 28–30, 32, 36, 50, 53, 57, 60, 64, 68, 69, 74]
Security	[19, 30, 44, 44, 57, 60, 66]
Ease of use	[27, 28, 36, 42, 60, 63, 68, 71]
Web based	[22, 24–26, 29, 30, 37, 44, 49–53, 55, 59, 63, 73]
Having a data warehouse	[27, 28, 30, 44, 60]

**Table 3:** Challenges with dashboards and solutions

Main challenges	Solutions
*Data sources and data generation*	
Combining multiple computerized systems	Entering the correct data
Different data formats	Create data warehouse
Data quality	Automatic data collection
Fragmented source systems	Use of web service architecture and middleware
Redundancy of the data	
Manual data entry	
*Dashboard content*	
Different needs of users	Stakeholder participation Identify performance indicators appropriate to the goals of the organization
The information displayed does not match the needs of users	
Non-compliance of key performance indicators with the goals of the organization	
Problems with measuring too many performance indicators	
Patient privacy	
*Dashboard design*	
Users’ differing executive duties	Use of customization feature
Cognitive abilities and analytical skills	Use of colour-coding system
Problem with type of information displayed through visualization tools	Use of visualization
Amount of information presented on dashboard	Accurate understanding of the capabilities of existing systems
Provide timely data	
*Implementation and integrating*	
Lack of compatibility with workflows	Use of data exchange standards
Context sensitivity	Security
Lack of integration with other hospital systems	User training
Service disruption	Gradual implementation
System security	
Accessing the dashboard	
Increased workload	

### Findings

**Fig. 1:**
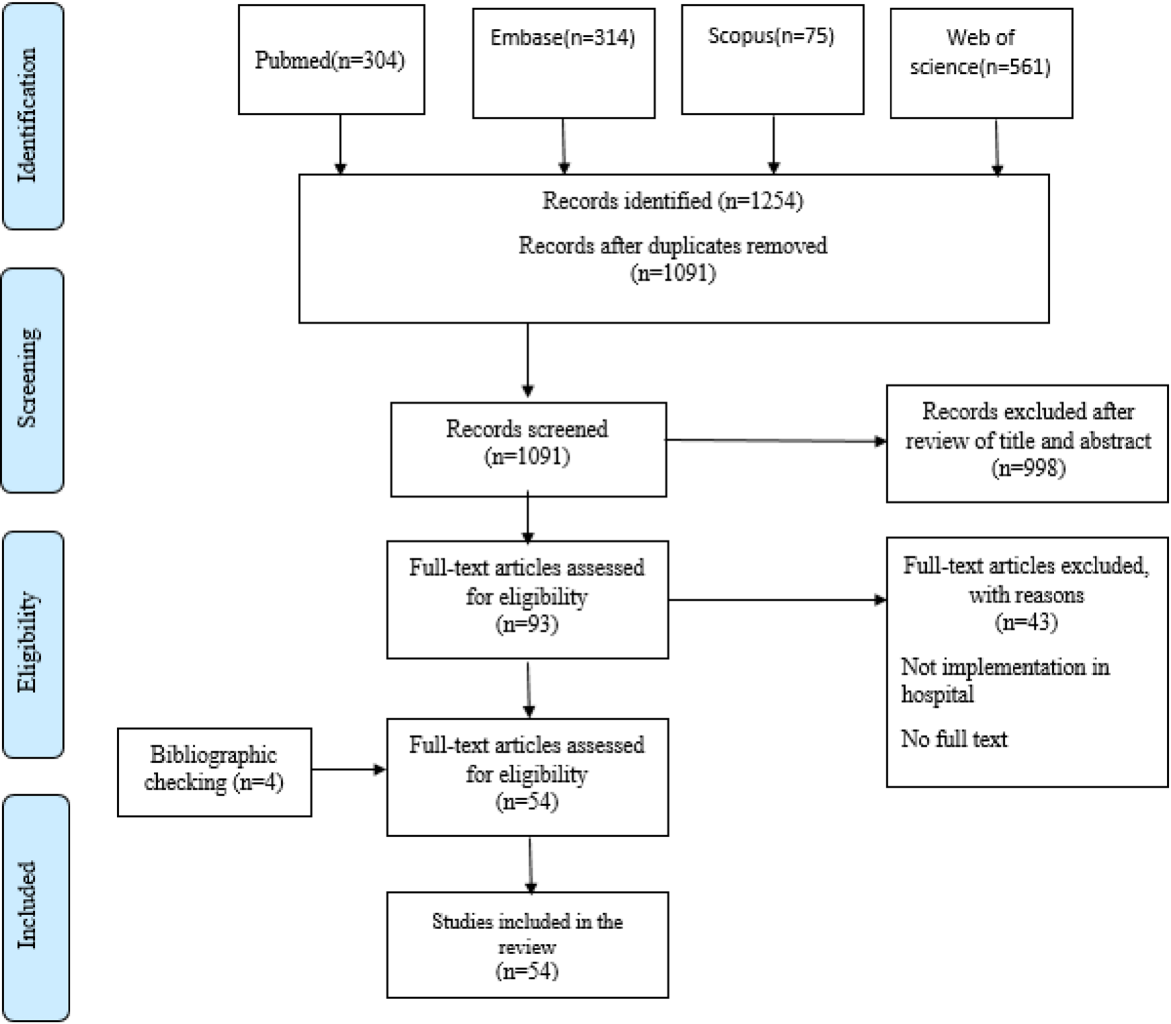
The flow diagram of the study based on the PRISMA statement

A total of 1254 articles were retrieved in this study, 163 of which were removed due to duplication. Of 1091 articles remaining, 998 were removed after reviewing their titles and abstracts (93 remaining articles). Next, the full-text manuscript of the articles was reviewed, resulting in the elimination of 43 articles. Finally, 50 articles were found eligible for this study. Besides, the reference lists of the selected articles were hand-searched, resulting in the identification of four studies. All extracted articles had an acceptable quality, and no study was removed due to low quality. Finally, a total of 54 articles were considered eligible and examined in this study. The article selection process is shown in Fig. 1.

### Quality assessment

According to the quality assessment of papers, 22 studies (41%) were considered as “high quality”; 12 studies (22%) were assigned as “fair to good quality”, and 20 studies (37%) were regarded as low quality (Additional file 1: Appendix A).

**Fig. 2:**
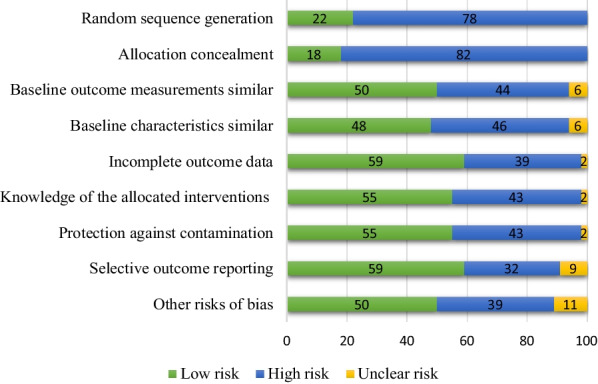
Percent of quality assessment criteria based on EPOC

As Fig. 2 shows, 82% of articles demonstrated a high risk of bias in relation to allocation concealment and 78% demonstrated a high risk of bias with respect to random sequence generation. In addition, 59% of articles showed a low risk of bias pertaining to incomplete outcome data and selective outcome reporting (Fig. 2).

### Characteristics of eligible studies

**Fig. 3:**
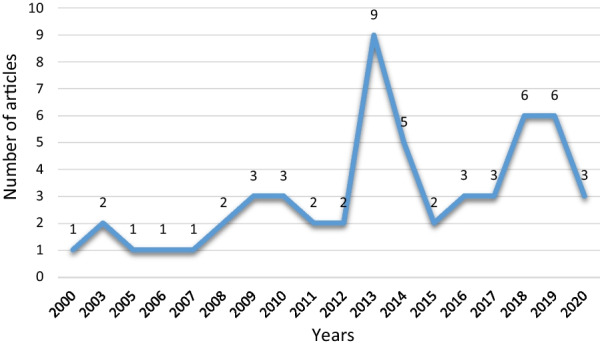
Number of publication by year

**Fig. 4:**
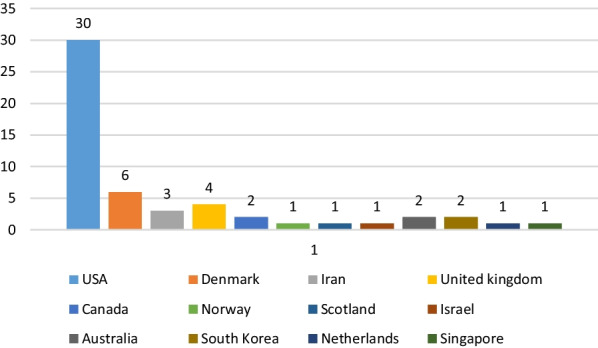
Number of publication by country

**Fig. 5:**
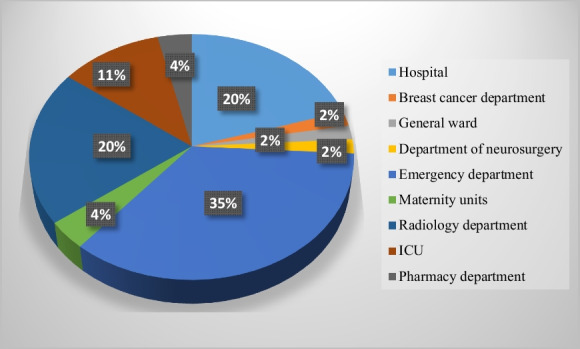
Number of publication by setting

Among the 54 studies, 32 were non-experimental studies (59%), of which 15 were descriptive studies, 14 were cross-sectional studies, and 3 were case studies. Twenty-two studies were experimental studies (41%), of which 21 were non-randomized studies and one was a randomized controlled study. As Fig. 3 shows, the use of dashboards in healthcare is increasing. The majority of eligible studies were performed in the USA, followed by Denmark, England, and Iran (Fig. 4). Most of these studies (n = 43) were conducted in different departments, while 11 studies were performed at hospital levels. Among hospital wards, the majority of studies were performed in the emergency department, followed by the radiology unit and the intensive care unit (Fig. 5).

### Hospital dashboard requirements

Table 2 lists the functional and non-functional goals and requirements of dashboards.

According to the reviewed papers, the goals of the dashboard include care quality assessment, resource management (staff and equipment), monitoring ongoing activities at the hospital level, and analyzing and forecasting hospital activities. Functional requirements for both quality and clinical dashboards in the literature include the following:

*Customization*: It enables users to change the type of indicators displayed by the dashboard and optimize their view of the content shown on the screen to best suit their needs and preferences. Customizability in quality dashboards can be used to display and monitor performance indicators that are important to the user and require real-time monitoring. In clinical dashboards, it serves to display clinical information essential to assessing patients’ status.

*Alerts creating*: This feature creates alerts for indicators that are necessary to the user and demand real-time monitoring. It also gives an alert when the value of the indicators exceeds the defined standard. Alert creation in quality dashboards is used when the value of indicators is higher than the defined standard. In clinical dashboards, this feature alerts physicians or nurses when the results of tests or imaging are ready.

*Tracking*: This feature tracks the location of patients for real-time monitoring of ongoing activities, identifying crowded wards, and proper allocation of resources. In quality dashboards, this feature is used to determine the location of patients and monitor crowded wards. In clinical dashboards, it serves to locate the physician's instructions and monitor them until the results are ready.

*Measuring performance indicators*: This involves comparing indicators with standards or with the national average, and comparing indicators over time. In quality dashboards, it is used to measure performance indicators, and in clinical dashboards, to display patients' clinical information.

*Reporting*: It is the ability to prepare visual reports based on clinical performance indicators in clinical dashboards, and based on managerial performance indicators in quality dashboards. It also involves the ability to create output files in various formats (Excel, Word, PDF),

*Reminders*: This feature creates reminders about the time of maintenance and inspection of hospital equipment.

### Challenges with dashboards and solutions

The identified challenges were categorized into four groups: data sources and data generation [26–30, 40, 47, 54, 56, 69, 71, 72], dashboard content [19, 31, 36, 41, 46–48, 50, 57, 59], dashboard design [19, 26, 33, 34, 36, 40, 41, 54, 69, 70], and implementation and integration [19, 26, 28, 33–37, 40, 44, 50, 56, 67, 69, 70, 72]. The proposed strategies to eliminate these challenges are presented in Table 3.

The proposed solutions to alleviate the challenges of identifying data sources and generating data include inputting the accurate data to the dashboard [44], creating a standard-format data warehouse for data manipulation to facilitate sharing and reduce processing time, updating and creating queries from the dashboard [27, 30, 44, 57], using the architecture of Web services and middleware [26, 29, 44], and utilizing automatic data extraction methods to solve the manual data input problem [47]. The proposed solutions to reduce the challenges of identifying the content of dashboards include stakeholder participation in the development, implementation, and evaluation of the dashboard [19, 31, 36, 44, 54, 63], identification of performance indicators suitable for organizational goals, and not selecting too many indicators for reporting by the dashboard [44]. The proposed solutions to mitigate the challenges associated with dashboard design are matching graphics to the purpose of the content, timely display of data, using a color-coding system, adding customization capabilities [19, 31, 32, 48, 49, 57, 63], proper organization and display of information using visualization tools to help users read and interpret information faster [44], accurate understanding of the capabilities of old systems and how they support clinical activities [34]. The proposed solutions to decrease the challenges of dashboard implementation and integration include integrating and linking the dashboard to other systems, and using data exchange standards [19, 26, 30, 33, 44, 51, 57], user training [36], paying attention to dashboard security when integrated with other systems, determining the access level based on user tasks [19, 44, 57, 60], and gradual implementation of the dashboard [36].

## Discussion

This systematic review aimed to identify the functional and non-functional requirements and challenges of using dashboards in hospital settings. Based on the findings, reporting, customization, reminders, assessment of performance indicators, alert creation, and tracking were identified as the functional requirements of quality/clinical dashboards [75, 76]. In a study by Buttigieg et al., monitoring, analysis, alerts, and color coding were described as the main functional requirements of clinical dashboards [7, 16]. Besides, Ghazi Saeedi et al. found alarms, drill down, and timely presentation as the central features of quality dashboards [77]. In Randell et al.'s study [78], the functional requirements of quality dashboards included visualization, interaction, data quality, reporting, and notification.

However, functional capabilities are used in both quality and clinical dashboards depending on the intended environment and purpose. Previous studies found speed, security, ease of use, and integration in other systems as the main non-functional features of dashboards [79]. The findings of the present study also indicated the importance of the mentioned features as non-functional requirements. Moreover, attention to the user-friendliness and user interface (UI) of the software, tailored to the needs of users, increases the successful implementation and continuous use of the system [80]; In the present study, features such as speed, security, and integration with other systems constitute the non-functional requirements of dashboards. To promote the user-friendliness of the system user interface, data visualization tools suited to the nature of data and users’ perception and knowledge should be employed.

In the current study, the identified challenges were categorized into four groups: data sources and data generation, dashboard content, dashboard design, and implementation and integration. In a study by Rasmussen et al., four types of challenges, including presentation format, integration, interface design, and development and implementation, were described [81]. Since the presentation format is related to the UI design [82], in the present study, it was classified as dashboard design.

Similarly, Ghazi Saeedi et al. reported four types of implementation challenges, including the development of performance indicators, data sources and data generation, integration of dashboards in source systems, and information presentation problems [77]. In the present study, the development of performance indicators was classified as dashboard content. Regarding the challenges of data sources, due to the dispersion of systems and storing data in different formats in these systems, the creation of a data warehouse for data storage and web service architecture is suggested. In addition, development of a data warehouse is one of the methods to prevent duplications in a dashboard [13, 81, 83].

According to previous studies, data availability is also a major prerequisite for dashboard development [12, 84]. Besides, a service-oriented architecture is necessary for encapsulating data from different systems in a middleware layer for data integration in dashboards, and understanding various data hosting structures, different methods of data proliferation and transfer, and the best query language are necessary for this data structure [85].

Regarding the challenges of dashboard content, in the present study, in relation to the type of information displayed by the dashboard and non-compliance with user needs, user participation and focus on selecting indicators appropriate to the goals of the organization is proposed. Evidence suggests the necessity of engaging users in dashboard development and adaptation processes to reduce resistance to the implementation of these systems [81].

Generally, it is important to select the type and number of indicators in a dashboard [86, 87], and every organization needs to select appropriate indicators depending on its goals [88]. Evidence shows that at least 15–25 indicators are essential in a dashboard [89].

Regarding the challenges of dashboard design, in the present study, to have a compatible system with the users’ cognitive abilities and skills and to provide information in a timely manner s, the capability of customization (to display information tailored to the user’s needs), color-coding systems, and visualization tools has been suggested. Overall, the findings of the current study were consistent with previous studies. According to previous studies, features, such as drill-down, filter, and alerts, are needed in dashboards for customizing the data depending on the user’s needs [12, 17, 90]. Overall, customization is an essential feature for organizing the dashboard content according to the users’ needs and promoting its application by the users [89, 91]. Besides, a color-coding system can be useful for a better understanding and interpretation of displayed information [12].

Besides, the reviewed studies had not used the same techniques to visualize performance indicators. Based on the present study, depending on the type of dashboard, its context, and users, a variety of interactive and visualization techniques are employed in dashboard design. Research suggests that using the same visualization model (in which visualization is performed without considering the user's preferences, abilities, or contexts) would not be effective. On the other hand, the development of adaptive and personalized visualization systems (using which users can change the type of information display according to their individual cognitive style and ability) will help better understand the information displayed by the dashboard [91, 92]. Moreover, in the dashboard, the nature of data and human factors, e.g., experience, skills, preferences, and cognitive styles influence the choice of visualization and interaction techniques [93, 94].

Additionally, regarding the challenges of dashboard implementation and integrating, the present study highlighted the importance of integrating data exchange standards, determining data access levels for dashboards, gradual adaptation of dashboards, and user training as potential solutions to facilitate dashboard adaptation and address its incompatibility with other systems. Since different hospital systems are integrated and linked to dashboards, particular attention should be paid to data security. In addition, data exchange standards are necessary for communication between systems and their integration in hospitals [95]. Theoretically, depending on the importance and quality of information in dashboards, the level of data accessibility can be controlled at different security/privacy levels [96]. It is also recommended to control the level of access based on the user's role and incorporate features, such as read-only or write/edit access [96, 98]. Moreover, the use of “single sign-on” technology is recommended for user login [98]. The present findings also indicated the significant contribution of gradual dashboard implementation and inclusion of features based on the users’ needs for achieving success and meeting the users’ needs [99].

It is also important to pay attention to the context when implementing the dashboard. For instance, a delay in reporting the results of a head CT scan at the emergency department is critical if it takes more than a few minutes; however, a delay in the same CT scan performed on an outpatient basis in other departments may not be important even if it takes several hours. Additionally, a radiologist may have hundreds of reports to sign per day, while an angiographer may have fewer than 10 reports. In the intensive care unit (ICU) and emergency department, the real-time feature is more important than in other departments. Because of these workflow determinants, dashboards must combine context-sensitive parameters to effectively manage workflows. Therefore, dashboards should be optimized, context-sensitive, customizable, and workflow-integrated.

### Strengths and limitations

A strength of this study was providing a comprehensive view of the functional and non-functional requirements of dashboards designed in hospitals. It also delineated and categorized the dashboard implementation challenges that may arise in dashboard design and implementation steps and offered solutions to overcome these challenges. The results of this study can lay the ground for better design and implementation of dashboards at the hospital level. It is important to address some limitations of this study. First, all retrieved articles were written in English. Second, data were extracted by one researcher (SA) and evaluated by two researchers (SA and RR), which probably resulted in the unintended removal of some eligible studies. Finally, the inclusion and exclusion criteria of this study focused on the implemented hospital-level dashboards, while those used for data management related to a particular disease were excluded.

### Future research

The present study revealed several issues in the evaluation and design of quality/clinical dashboards. Studies should be conducted to examine the effectiveness of using quality/clinical dashboards in patient care processes, to assess the workload of users when using the dashboard, and to measure situation awareness in the presence of the dashboard. Studies should also focus on interactive techniques and data visualization in quality and clinical dashboards according to the environment in which they are used.

## Conclusion

As a data management tool, dashboards play a significant role in managing significant amounts of data in healthcare settings, including hospitals. According to the findings, functional requirements for a hospital dashboard were as follows: reporting, customization, reminders, assessment of performance indicators, alert creation, and tracking. Also, non-functional indicators included speed, security, ease of use, installation on different devices (e.g., PCs and laptops), integration in other systems, web-based design, inclusion of a data warehouse, being up-to-data, and use of data visualization elements based on the users' needs; these features contribute to the adaptation and success of dashboards. Considering the challenges of dashboard implementation in hospital settings, particular attention needs to be paid to data sources, dashboard content, UI design, and dashboard implementation and integration in other hospital systems. Finally, by examining the functional and non-functional requirements of hospital dashboards, and by enumerating the various challenges in dashboard design and implementation, these results can be a basis for improving the design and implementation of dashboards at the hospital level.

## Supplementary Information

**Additional file 1: Appendix A**. The result of qualitative evaluation of studies.

## Data Availability

All data used in the publication of this work were obtained from published studies. The abstracts for these studies are available in the web of science, pubmed, embase, and scopus database.
