# Supplementary material for: Requirements and challenges of hospital dashboards: a systematic literature review
Source: BMC Med Inform Decis Mak. 2022 Nov 8;22:287. doi: 10.1186/s12911-022-02037-8 (PMC9644506; doi:10.1186/s12911-022-02037-8)
Supplement: Supplementary file 1 — Additional file 1: Appendix A. The result of qualitative evaluation of studies. [file 12911_2022_2037_MOESM1_ESM.docx]

**Additional file 1: Appendix A: The result of qualitative evaluation of studies**

| Author | 1 | 2 | 3 | 4 | 5 | 6 | 7 | 8 | 9 | Quality assessment |
| --- | --- | --- | --- | --- | --- | --- | --- | --- | --- | --- |
| Weggelaar-Jansen et al. (18) | H | H | U | H | H | H | H | H | H | Low quality |
| Seltzer et al. (22) | H | H | H | H | H | H | H | L | H | Low quality |
| Boger (23) | H | H | H | H | L | L | L | L | U | Fair to Good quality |
| Nagy et al. (24) | H | H | U | U | L | L | U | U | L | Fair to Good quality |
| France et al. (25) | H | H | L | L | L | L | L | L | L | High quality |
| Morgan et al. (26) | H | H | H | H | H | H | H | H | H | Low quality |
| Bahl et al. (27) | H | H | H | H | L | H | H | H | H | Low quality |
| Aronsky et al. (28) | H | H | H | H | H | H | H | H | H | Low quality |
| Morgan et al. (29) | H | H | L | H | H | H | H | H | H | Low quality |
| Nagy et al. (30) | H | H | L | H | L | L | L | U | U | Fair to Good quality |
| Wong et al. (31) | H | H | L | L | L | L | L | L | L | High quality |
| Zaydfudim et al. (32) | L | H | L | L | L | L | L | L | L | High quality |
| Abujudeh et al. (33) | H | H | H | L | H | L | H | U | U | Fair to Good quality |
| Bisantz et al. (34) | H | H | L | L | L | H | H | L | L | Fair to Good quality |
| Rasmussen et al. (35) | H | H | H | U | H | L | L | L | L | Fair to Good quality |
| Hertzum (36) | L | H | L | L | L | L | L | L | L | High quality |
| Waitman et al. (37) | H | H | H | H | L | L | L | L | L | Fair to Good quality |
| Sebastian et al. (38) | H | H | H | H | H | H | H | H | H | Low quality |
| Stone-Griffith et al. (39) | L | L | L | L | L | L | L | L | L | High quality |
| Daley et al. (40) | H | H | H | H | H | H | H | H | H | Low quality |
| Dexheimer and Kennebeck. (41) | H | H | H | H | H | H | H | H | H | Low quality |
| Dolan et al. (42) | H | H | H | L | L | L | L | L | L | High quality |
| Hertzum and Simonsen. (43) | L | L | L | L | L | L | L | L | L | High quality |
| Karami et al. (44) | H | H | H | H | H | H | H | H | H | Low quality |
| Koch et al. (45) | L | L | L | L | L | L | L | L | L | High quality |
| Mansoori et al. (46) | H | H | L | H | H | L | L | L | L | Fair to Good quality |
| Simms et al. (47) | L | L | L | L | L | L | L | L | L | High quality |
| Sprague et al. (48) | L | L | L | U | H | H | L | L | U | Fair to Good quality |
| Crofts et al. (49) | H | H | H | H | L | H | H | H | H | Low quality |
| Gjaere and Lillebo. (50) | L | L | L | L | L | L | L | L | L | High quality |
| McLaughlin et al. (51) | H | H | H | L | L | H | H | U | U | Fair to Good quality |
| Russell et al. (52) | H | H | H | H | H | H | H | H | H | Low quality |
| Swartz et al. (53) | H | H | U | L | U | U | L | U | U | Fair to Good quality |
| Hartzler et al. (54) | H | L | L | L | L | L | L | L | L | High quality |
| Shaw et al. (55) | L | H | L | L | L | L | L | L | L | High quality |
| Hertzum and Simonsen. (56) | H | H | L | L | L | L | L | L | L | High quality |
| Karami and safdari. (57) | H | H | H | H | H | H | H | H | H | Low quality |
| Mazor et al. (58) | H | H | H | H | H | H | H | H | H | Low quality |
| Franklin et al. (59) | H | H | H | H | H | H | H | H | H | Low quality |
| Lee et al. (60)L | H | H | L | H | L | L | L | L | H | Fair to Good quality |
| Staib et al. (61) | H | H | H | H | H | H | H | H | H | Low quality |
| Martin et al. (62) | H | H | H | H | L | H | H | L | H | Low quality |
| Martinez et al. (63) | H | H | H | H | H | H | H | H | H | Low quality |
| Romero-Brufau et al. (64) | H | H | L | L | L | L | L | L | L | High quality |
| Shailam et al. (65) | L | L | L | L | L | L | L | L | L | High quality |
| Yoo et al. (66) | H | H | L | L | L | L | L | L | L | High quality |
| Hester et al. (67) | H | H | L | L | L | L | L | L | L | High quality |
| Jebraeily et al. (68) | H | H | H | L | H | H | H | H | H | Low quality |
| Randell et al. (69) | H | H | L | H | L | L | L | L | L | High quality |
| Schmidt et al. (70) | H | H | H | H | H | H | H | H | H | Low quality |
| van Deen et al. (71) | H | H | L | L | L | L | L | L | L | High quality |
| Dixit et al. (72) | H | H | L | L | L | L | L | L | L | High quality |
| Janssen et al. (73) | L | L | L | L | L | L | L | L | L | High quality |
| Williams et al. (74) | L | L | L | L | L | L | L | L | L | High quality |
| Low risk n (%) | 12(22) | 10(18) | 27(50) | 26(48) | 32(59) | 30(55) | 30(55) | 32(59) | 27(50) |  |
| High risk n (%) | 42(78) | 44(82) | 24(44) | 25(46) | 21(39) | 23(43) | 23(43) | 17(32) | 21(39) |  |
| Unclear risk n (%) | - | - | 3(6) | 3(6) | 1(2) | 1(2) | 1(2) | 5(9) | 6(11) |  |

L; Low risk H; High risk U; Unclear risk

1; Random sequence generation

2; Allocation concealment

3; Baseline outcome measurements similar

4; Baseline characteristics similar

5; Incomplete outcome data

6; Knowledge of the allocated interventions adequately prevented during the study

7; Protection against contamination

8; Selective outcome reporting

9; Other risks of bias
